# Human cytomegalovirus inhibits apoptosis by regulating the activating transcription factor 5 signaling pathway in human malignant glioma cells

**DOI:** 10.3892/ol.2014.2264

**Published:** 2014-06-18

**Authors:** TONGMEI WANG, DONGMENG QIAN, MING HU, LING LI, LI ZHANG, HAO CHEN, RUI YANG, BIN WANG

**Affiliations:** Department of Microbiology, Qingdao University Medical College, Qingdao, Shandong 266071, P.R. China

**Keywords:** glioblastoma cells, activating transcription factor 5, apoptosis, human cytomegalovirus infection

## Abstract

The activating transcription factor 5 (ATF5), also termed ATFx, is a member of the ATF/cAMP response element-binding protein (CREB) family of basic zipper proteins. ATF5 is an anti-apoptotic protein that is highly expressed in malignant glioma and is essential for glioma cell survival. Accumulating evidence indicates that human malignant gliomas are universally infected with human cytomegalovirus (HCMV). Recent studies have shown that HCMV may be resistant to the induction of apoptosis by disrupting cellular pathways in glioblastoma. To investigate the potential anti-apoptotic function of HCMV in glioma, malignant U87 glioma cells were infected with HCMV. The present study showed that HCMV infection suppressed apoptosis in glioblastoma U87 cells by regulating the expression of ATF5. Furthermore, in glioblastoma U87 cells, HCMV infection induced cellular proliferation in parallel with an increase in the expression level of ATF5 and B-cell lymphoma/leukemia-2 to Bcl-2-associated X protein ratio. Loss of ATF5 function was achieved using a dominant-negative form of ATF5 in U87 cells, whereby cells appeared to grow marginally following HCMV infection when compared with the control. However, the anti-apoptotic ability was appeared to decline in the terminal deoxynucleotidyl transferase-mediated dUTP nick end labeling assay. These results indicate that ATF5 signaling pathways may be important in the anti-apoptotic activity of HCMV-infected glioblastoma cells; therefore, the anti-apoptotic molecular mechanisms of HCMV in human glioblastoma cells were investigated in the current study. Prevention of HCMV infection may present a potential and promising approach for the treatment of malignant gliomas.

## Introduction

Glioblastomas are a particularly devastating form of primary brain tumor. Due to the highly infiltrative and invasive nature of such tumors, it remains clinically intractable. Patient survival time is generally only 12–18 months even following surgical resection with subsequent radiation and chemotherapy (1–3). Recent studies have indicated that the development of a tumor is often associated with the regulation of a variety of transcription factors (1,4,5). Activating transcription factor 5 (ATF5) is a novel factor that is closely associated with tumor cell differentiation, proliferation and apoptosis. ATF5 is a member of the ATF/cAMP responsive element-binding (CREB) family of transcription factors, which includes a large group of basic leucine zipper proteins that mediate diverse transcriptional regulatory functions (6–9). ATF5, an anti-apoptotic factor, is highly expressed in malignant glioma and is important in the promotion of cell survival (5). ATF5 loss of function induces apoptosis in a number of glioma and breast cancer cell lines (10,11), however, interfering with ATF5 function in non-tumor brain cells has not been found to affect their survival (10). Dluzen *et al* (12) demonstrated that B-cell lymphoma/leukemia-2 (Bcl-2) is a downstream target of ATF5 in gliomas and breast cancer. The Bcl-2 family of proteins includes anti-apoptotic proteins, such as Bcl-2, Bcl-Xl and induced myeloid leukemia cell differentiation protein, and apoptotic proteins, such as Bcl-2 homologous antagonist/killer, Bcl-2 associated X protein (BAX), BH3 interacting-domain and B-cell lymphoma 2 interacting mediator of cell death. The regulation and balance of the Bcl-2 family proteins in a particular cell results in the inhibition or induction of apoptotic signaling pathways (12–14).

The human cytomegalovirus (HCMV) infection has been detected in malignant gliomas in a high percentage of cases, although not in the adjacent healthy brain tissues (15). Growing evidence indicates that HCMV infection may increase the malignancy of infected cells by disrupting cellular pathways, such as apoptosis (16–18). Apoptosis is detrimental to HCMV, as it functions as a cellular antivirus response to eliminate infected cells (by activating the immune response) or is deleterious (an inevitable consequence of the stress that is inflicted by viruses on host cells). To survive, these viruses have developed numerous strategies to prevent the premature cell death of host cells (19,20). HCMV infection in glial cells that does not lead to cell apoptosis may promote clonal expansion without producing a productive or cytopathic virus infection. Long-term persistence of HCMV in malignant glioma cells may result in the occurrence of variant strains, which exhibit a minimal cytopathic effect, and therefore, HCMV may be reactivated in latently infected glioma cells when cells are exposed to inflammatory stimuli or superinfected with other HCMV strains (21,22). The sustained expression of specific HCMV gene products may promote the overall glioma phenotype, as HCMV encodes for gene products that regulate cellular pathways involved in mutagenesis and apoptosis, and host antitumor immune responses (23). HCMV immediate-early (IE) genes 1 and 2 are the first set of viral genes that are activated within HCMV-infected cells (24). IE1 and IE2 proteins regulate transcription of viral and cellular genes within HCMV-infected cells (25). In addition, the IE protein has a binding site for the ATF/CREB family of transcription factors, which upon binding forms a complex to activate downstream elements (26).

Due to the high prevalence of HCMV and ATF5 expression observed *in vivo* in human malignant glioma, the aim of the present study was to investigate the role of the ATF5 signaling pathway in HCMV-infected glioblastoma cells.

## Materials and methods

### Cell lines and viruses

Human glioblastoma U87 cell lines were purchased from the Shanghai Cell Resource Center of the Chinese Academy of Sciences (Shanghai, China). U87 cells were propagated in HyClone^TM^ Minimum Essential Medium with 10% fetal bovine serum (Thermo Fisher Scientific Inc., Rockford, IL, USA) and maintained at 37°C in a humidified atmosphere containing 5% (v/v) CO_2_. HCMV AD169 (France Pasteur Laboratory, Paris, France) was tittered by plaque titration in human embryonic lung fibroblast cells and expressed as the number of plaque-forming units per milliliter. The HCMV was propagated in human embryonic lung fibroblast cells with serum-free medium and the cell supernatant was harvested and stored at −80°C.

### Loss of ATF5 expression or function achieved using small interfering (si) RNA or a dominant-negative (dn) form of ATF5

To establish U87 cell lines with a stable knockdown of ATF5, a GV113 control plasmid and three GV113 plasmids containing ATF5 lentivirus short hairpin RNAs, LV-ATF5-RNAi (8842-1; GCGAGATCCAGTACGTCAA), LV-ATF5-RNAi (8843-1; TCTTGGATACTCTGGACTT) and LV-ATF5-RNAi (8844-1;TGGAACAGATGGAAGACTT) (Shanghai GeneChem Co., Ltd., Shanghai, China), targeting the ATF5 coding sequence were separately transduced into U87 cells.

The pLeGFP-C1-NTAzip-ATF5 plasmid was transfected to block the function of ATF5 simultaneously. Cells were transfected with pLeGFP-C1-NTAzip-ATF5 and the pLeGFP-C1 plasmid using Lipofectamine 2000 (Invitrogen Life Technologies, Carlsbad, CA, USA). To analyze proliferation and apoptosis and to perform western blot assays, cells were collected at 0, 12, 24 and 48 h following transfection.

### 3-(4,5-Dimethylthiazol-2-yl)-2, 5-diphenyltetrazolium bromide (MTT) assay

Cell proliferation was determined using the MMT assay (Sigma-Aldrich, St. Louis, MO, USA). Glioblastoma U87 cells were plated in 96-well microplate formats according to the manufacturer’s instructions. Cell lines were seeded in five replication wells at 5,000 cells/well and cultured for 0, 12, 24 or 48 h. Following MTT uptake for a duration of 4 h, cells were lysed in 150 μl dimethyl sulfoxide and absorbance was measured at 475 nm using a fluorescence microplate reader (Sunrise Remote, Tecan Austria GmbH, Grödig, Austria).

### RNA extraction, and quantitative polymerase chain reaction (qPCR)

RNA was extracted using TRIzol reagent [Takara Biotechnology (Dalian) Co., Ltd., Dalian, China] according to the manufacturer’s instructions. To produce cDNAs, 1 μg RNA was reverse-transcribed using the PrimeScript RT reagent kit with gDNA eraser (Perfect Real Time; Takara Biotechnology [Dalian] Co., Ltd.) according to the manufacturer’s instructions. qPCR analyses were performed using the GoTaq qPCR Master Mix (Promega Corporation, Madison, WI, USA). The following primers were used: Forward, 5′-AGTGGGCTGGGATGGCTCGTAGAC-3′ and reverse, 5′-CTCGGGTGGTGGCAGGATGTGG-3′ for ATF5; forward, 5′-GCGCAATATCATGAAAGATAAGAACA-3′ and reverse, 5′-GATTGGTGTTGCGGAACATG-3′ for IE2; forward, 5′-CTGCACCTGACGCCCTTCACC-3′ and reverse, 5′-CACATGACCCCACCGAACTCAAAGA-3′ for Bcl-2; forward, 5′-TGGAGCTGCAGAGGATGATTG-3′ and reverse, 5′-GAAGTTGCCGTCAGAAAACATG-3′ for BAX; and forward, 5′-TGGAACGGTGAAGGTGACAG-3′ and reverse, 5′-GGCTTTTAGGATGGCAAGGG-3′ for β-actin.

### Western blot analysis

Cells were washed three times with ice-cold phosphate-buffered saline (PBS). Next, cells were dissolved in 500 μl lysis buffer and 5 μl phenylmethylsulfonyl fluoride (Beyotime Institute of Biotechnology, Shanghai, China). Samples were centrifuged (Eppendorf 5804R, Eppendorf Corporation, Hamburg, Germany) at 20,100 × g for 5 min at 4°C to separate the membrane fraction from the cytosolic fraction. After boiling for 5 min, equivalent amounts of protein (30 μg) were resolved by 12% SDS-PAGE, electroblotted onto polyvinylidene fluoride membrane (Millipore, Billerica, MA, USA) and immunoreacted overnight with IE genes (Virostat, Inc., Westbrook, ME, USA), ATF5 (Abcam, Cambridge, UK), Bcl-2 and Bax (Bioss, Inc., Woburn, MA, USA), followed by a 2-h incubation with the horseradish peroxidase-conjugated secondary antibody (Bioss, Inc.). Chemiluminescent signals were generated by the SuperSignal West Pico Trial Kit (Thermo Fisher Scientific Inc.) and detected using the Vilber Lourmat imaging system (Vilber Lourmat Corporation, Torcy, France).

### Terminal deoxynucleotidyl transferase-mediated dUTP nick end labeling (TUNEL) assay

To detect the apoptosis rate of HCMV-infected glioblastoma cells, an *in situ* cell death detection kit (Roche Diagnostics Corporation, Indianapolis, IN, USA) was used. Cells grown in 6-well culture clusters were treated with HCMV for 48 h. Treated cells were fixed onto poly-(L-lysine) coated slides with 4% paraformaldehyde. The slides were rinsed with PBS and the cells were permeabilized with 0.1% Triton X-100 [Sangon Biotech (Shanghai) Co., Ltd., Shanghai, China]. Then, the slides were washed with PBS and the cells were incubated in 50 μl TUNEL reaction mixture for 60 min at 37°C in the dark. Next, 50 μl converter-POD (Roche, Basel, Switzerland) was added and incubated for 30 min at 37°C in a humidified chamber. Slides were rinsed with PBS. Next, 50 μl 3,3′-diaminobenzidine substrate was added and incubated for 10 min at 20°C. A total of 50 μl hematoxylin was then added and incubated for 3 min at room temperature. The slides were mounted under a glass coverslip with PBS and analyzed under a light microscope (Motic B1-223A, Motic Deutschland GmbH, Wetzlar, Germany).

### Statistical analysis

Data are presented as the mean ± standard deviation. Statistical analysis was performed using the Student’s t-test and P<0.05 was considered to indicate a statistically significant difference.

## Results

### Effect of HCMV infection on cell proliferation and expression of ATF5, Bcl-2 and BAX

**Figure 1:**
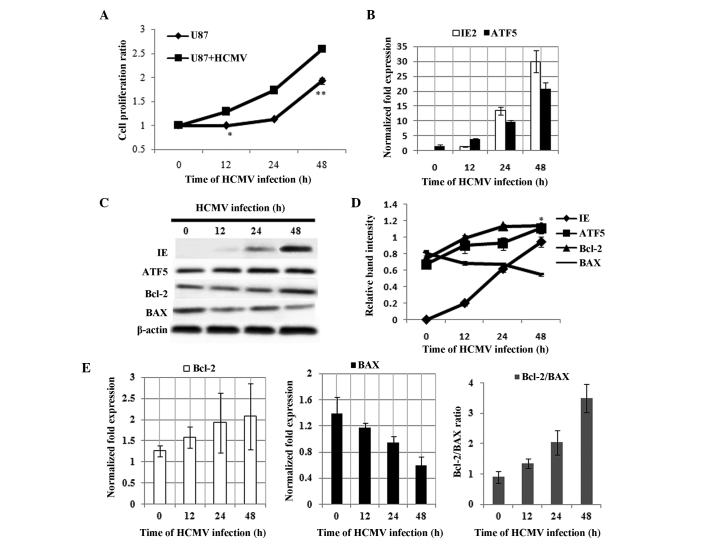
Effect of HCMV infection on cell proliferation and expression of ATF5, Bcl-2 and BAX in U87 cells. (A) Cell proliferation was measured using a 3-(4,5-dimethylthiazol-2-yl)-2,5-diphenyltetrazolium bromide assay. Data are presented as the mean ± SD of three independent experiments (^*^P<0.05 and ^**^P<0.01 vs. U87 + HCMV). (B) qPCR analyses showing the expression levels of IE2 and ATF5 mRNA in U87 cells following HCMV infection for 0, 12, 24 and 48 h. (C) Western blot analysis of IE genes, ATF5, Bcl-2 and BAX protein in HCMV-infected U87 cells. (D) Relative expression of IE, ATF5, Bcl-2 and BAX, all vs. β-actin in U87 cells infected with HCMV for 0, 12, 24 and 48 h, according to the results of figure 1C. Data are presented as the mean ± SD (^*^P<0.01 vs. 0 h ATF5 expression). (E) qPCR analyses of Bcl-2 and BAX mRNA expression in U87 cells following HCMV infection, where the ratio of Bcl-2/BAX was calculated. HMCV, human cytomegalovirus; ATF5, activating transcription factor 5; Bcl-2, B-cell lymphoma/leukmia-2; BAX, Bcl-2-associated X protein; SD, standard deviation; qPCR, quantitative polymerase chain reaction; IE, immediate-early.

To determine the role of ATF5 expression in the HCMV-infected U87 glioblastoma cells, the proliferation ratio of U87 cells was examined in response to HCMV infection. Compared with normal U87 cells, proliferation was enhanced by HCMV infection (Fig. 1A). This observation indicated that HCMV infection promotes growth proliferation in U87 glioblastoma cells. To elucidate the underlying mechanisms, the expression of ATF5 in HCMV-infected U87 cells was examined. As shown in Fig. 1B, ATF5 mRNA was upregulated 12 h following HCMV infection and continued to increase until 48 h following infection. Similarly, ATF5 protein levels were upregulated (Fig. 1C and D; P<0.05). These results demonstrated that HCMV infection upregulates ATF5 expression in U87 cells. Studies have shown that anti-apoptotic Bcl-2 is regulated by ATF5 in glioblastoma cells and BAX is an apoptosis member of the Bcl-2 family. To examine the expression of Bcl-2 and BAX in HCMV-infected U87 cells, qPCR and western blot analysis were performed. As shown in Fig. 1C–E, the ratio of Bcl-2 to BAX increased in U87 cells treated with HCMV (P<0.05). In addition, Bcl-2 protein levels were upregulated in U87 cells treated with HCMV (Fig. 1C and D; P<0.05). These results indicated that HCMV infection enhances the anti-apoptotic ability of U87 cells.

### Interfering with ATF5 affects HCMV-infected U87 cell proliferation

**Figure 2:**
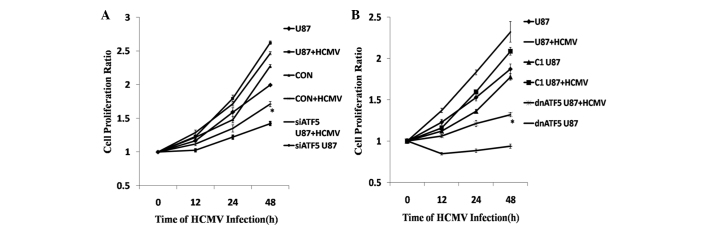
Cell viability analysis of siATF5 or dnATF5 U87 cells following HCMV infection. (A) U87 cells expressing non-targeting siRNA or ATF5 short hairpin RNA were infected with HCMV for 0, 12, 24 or 48 h. Knocking down ATF5 expression reduced tumor cell proliferation. HCMV-infected siATF5 U87 marginally promoted proliferation. (B) U87 cells were produced with loss of ATF5 function using a dn form. The results were consistent with siATF5 U87 cells. Cell proliferation was measured using 3-(4,5-dimethylthiazol-2-yl)-2,5-diphenyltetrazolium bromide assay. Data are presented as the mean ± standard deviation of three independent experiments (^*^P<0.05 vs. con + HCMV). si, small interfering; dn, dominant-negative; ATF5, activating transcription factor 5; HCMV, human cytomegalovirus; con, control.

To investigate the function of ATF5 in HCMV-infected U87 cells, the effect of silencing ATF5 on the proliferation of HMCV-infected U87 cells was examined. U87 cells were infected with lentiviral RNAi to interfere with ATF5. Western blot analysis revealed that ATF5 protein levels were markedly decreased (data not shown). The cells were subsequently infected with HCMV at various time points and cell proliferation was determined by MTT assay. As shown in Fig. 2A, compared with U87 cells, cell proliferation was reduced following ATF5 interference in U87 cells (P<0.05; between12 and 48 h). Cell viability following ATF5 interference in U87 cells was marginally increased following HCMV infection when compared with siATF5 U87 cells. To further validate this observation, U87 cells were transfected with dnATF5 plasmids to interfere with ATF5. As shown in Fig. 2B, the results were consistent with Fig. 2A. These results indicated that ATF5 may be involved in the regulation of cell proliferation of HCMV-infected U87 cells *in vitro*.

### Analysis of apoptosis in dnATF5 U87 treated with HCMV using TUNEL

**Figure 3:**
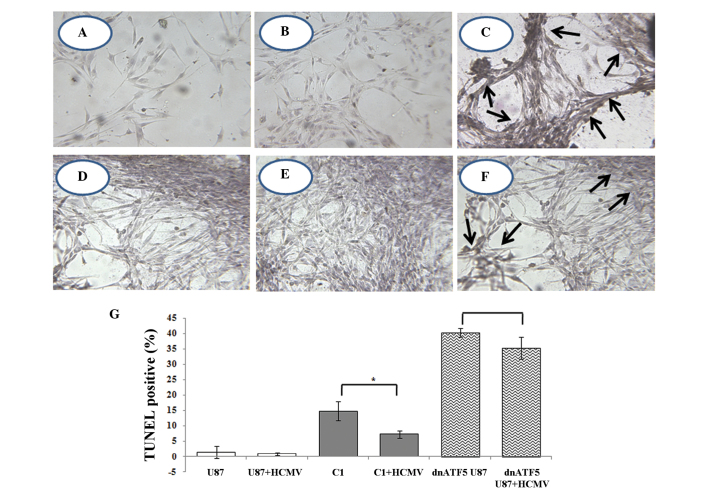
Apoptotic analysis of U87 cells exhibiting a loss of ATF5 function and treatment with or without HCMV by TUNEL assay and hematoxylin staining. (A) U87 cells and (B) HCMV-infected U87 cells did not demonstrate apoptosis. (C) Interfering with ATF5 function resulted in U87 cell apoptosis (as shown by arrows) and this data was consistent with previous studies (31). (D) U87 cells were transfected with pLeGFP-C1 plasmids using Lipofectamine 2000. A significant decrease was observed in (E) HCMV-infected control and TUNEL-positive cells. (C) U87 cells were transfected with the pLeGFP-C1-NTAzip-ATF5 plasmid and apoptosis was evident. (F) HCMV-infected U87 cells with ATF5 loss of function exhibited a decrease in TUNEL-positive cells. Magnification, 400. (G) Bar chart showing the mean apoptosis index per slide (A-F;^*^P<0.05). ATF5, activating transcription factor 5; HMCV, human cytomegalovirus; dn, dominant-negative; TUNEL, terminal deoxynucleotidyl transferase-mediated dUTP nick end labeling.

Previous studies have demonstrated that HCMV may increase the malignancy of glioma by blocking apoptosis. ATF5 is closely associated with tumor cell apoptosis. In addition, the present study found that HCMV infection regulates the expression of ATF5 in glioma. It is hypothesized that anti-apoptosis of HCMV infection may be associated with ATF5 pathways. In siATF5 U87 cells, almost no apoptotic cells were identified. However, apoptosis occurred in U87 cells, which were transfected with dnATF5 plasmids. Furthermore, apoptotic cell death was detected in ~40% of dnATF5 U87 cells. The number of TUNEL-positive cells (dead cells) decreased by 4–5% following HCMV infection. As shown in Fig. 3, TUNEL-positive cells were not observed in normal or U87 cells (Fig. 3A and B), however, TUNEL-positive cells were detected in dnATF5 U87 cells (Fig. 3C). Following HCMV infection for 48 h, 40% of TUNEL-positive cells were detected in dnATF5 U87 cells whereas 35% of TUNEL-positive cells were detected in HCMV-infected dnATF5 cells (Fig. 3G). Compared with the control, in U87 cells, which had lost ATF5 function, no significant difference was identified in the decline of TUNEL-positive cells (Fig. 3G). These results support the hypothesis that the anti-apoptotic effect of HCMV infection is associated with ATF5 expression.

### Bcl-2 and BAX expression following interference with ATF5 in HMCV-treated U87 cells

**Figure 4:**
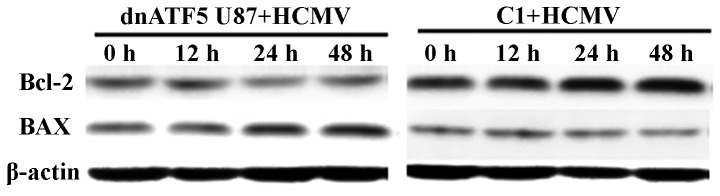
Western blot analysis showing changes in Bcl-2 and BAX protein levels in dnATF5 U87 cells treated with HCMV. The expression of the Bcl-2 and BAX protein was not altered following HCMV infection between 0 and 48 h in dnATF5 U87 cells when compared with the control. dnATF5, dominant-negative activating transcription factor 5; Bcl-2, B-cell lymphoma/leukmia-2; BAX, Bcl-2-associated X protein; HMCV, human cytomegalovirus.

Bcl-2 is a downstream target of ATF5. Interaction of BAX and Bcl-2 is important in the regulation of apoptosis. In the present study, it was found that HCMV infection in U87 cells regulated the expression of ATF5. Furthermore, following interference with ATF5 in U87 cells, the anti-apoptotic ability was decreased following HCMV infection. The expression of anti-apoptotic Bcl-2 and apoptotic BAX protein was further detected in HCMV-infected dnATF5 U87 cells by western blot analysis. In dnATF5 U87 cells, changes in Bcl-2 and BAX protein expression following HCMV infection are shown in Fig. 4. Compared with the control group, in HCMV-infected U87 cells, which had lost ATF5 expression, no significant differences in Bcl-2 and BAX protein levels between 0 and 48 h were identified.

## Discussion

Increasing evidence implicates infectious agents as causal factors in the development of human cancers. Infectious agents may be promoters of neoplastic transformation. For example, HCMV may modify host cell transcription to prolong its replication by establishing a latent infection. However, the modification may cause significant morbidity of cell life. Glioblastoma multiforme (GBM) is a malignant and lethal brain cancer of unknown origin, and the majority of cases are resistant to radiotherapy and chemotherapy (27). Over the past decade, various studies have searched for the presence of HCMV in GBM samples (23) and Cobbs *et al* (15) initially reported the expression of HCMV proteins and oligonucleotides in a high percentage of gliomas. Growing evidence that HCMV is specifically detected in a variety of human malignancies at low levels of expression indicates that the virus may facilitate the neoplastic process in malignancy via oncomodulation. In addition, oncomodulation implies that HCMV infects established tumor cells and increases their malignant potential without necessarily being oncogenic (16). Therefore, it is important to understand how this virus modifies the host.

Viruses have acquired the capacity to modify the host environment to enable the successful completion of their life cycle. The use and control of the cellular transcriptional machinery are among the most important functions of viruses. For example, apoptosis of infected cells may limit viral replication and thus serves as an innate defense mechanism against viral infection. Consequently, viruses delay apoptosis by affecting anti-apoptotic proteins. Recent studies have confirmed that HCMV establishes a replication-favorable environment to avoid prematurely compromising the cell’s ability to produce viral progeny (28). In addition, our previous studies have also confirmed that HCMV infection inhibits tumor necrosis factor-α induced apoptosis (29).

The present study revealed that HCMV infection blocks apoptosis in glioblastoma U87 cells and increases the expression levels of the ATF5 and Bcl-2 to BAX ratio. ATF5, a member of the ATF/CREB family of basic leucine zipper proteins, is an anti-apoptotic protein, which is highly expressed in malignant glioma, but not in normal brain tissues, and is essential for the survival of glioma cells. Our previous studies demonstrated that ATF5 is highly expressed in epithelial ovarian carcinomas and human pancreatic carcinomas, compared with healthy ovarian and pancreatic tissues (30,31). Although ATF5 is critically involved in cell survival, cell proliferation and differentiation, the upstream mechanism that regulates ATF5 function remains unclear.

The results of the current study revealed that the ATF5 signaling pathway is involved in the anti-apoptotic effects that are induced by HCMV infection in U87 cells. Previous studies have shown that ATF5 is an important anti-apoptotic protein in malignant glioma. However, to the best of our knowledge, no studies have shown that the anti-apoptotic effect of HCMV infection in malignant glioma is associated with ATF5. The results of the present study demonstrated that HCMV infection in U87 cells promotes cell proliferation and upregulates the expression of ATF5. In addition, when interfering with ATF5 in U87 cells, the anti-apoptotic ability was decreased following HCMV infection (Fig. 3). In revealing the anti-apoptotic mechanism of ATF5 in HCMV-infected glioma, it was found that Bcl-2 was downregulated in the U87 cells in which ATF5 was inhibited, when compared with the control (Fig. 4). Furthermore, previous studies have shown that Bcl-2 is a downstream target of ATF5 that mediates the pro-survival function of ATF5 in C6 glioma and MCF-7 breast cancer cells. ATF5 binds to an ATF5-specific regulatory element that is downstream of and adjacent to the negative regulatory element in the Bcl-2 P2 promoter, stimulating Bcl-2 expression (12) and the results of the present study were consistent with this. These results provide a novel insight into the influence of HCMV infection on tumor cell apoptosis.

In conclusion, the present study found that ATF5 is involved in the anti-apoptotic effect induced in HCMV-infected U87 cells. HCMV infection promotes the expression of ATF5 and elevates the Bcl-2 to BAX ratio and enhances anti-apoptosis in U87 cells. Furthermore, ATF5 knockdown may reduce the anti-apoptotic ability of HCMV-infected U87 cells. These results facilitate the understanding regarding the influence of HCMV infection on glioma cell apoptosis and thus, HCMV may present a potential therapeutic target.

## Abbreviations

ATF5: activating transcription factor 5
Bcl-2: B-cell lymphoma/leukmia-2
BAX: Bcl-2-associated X protein
CREB: cAMP response element-binding protein
HCMV: human cytomegalovirus
IE: immediate early
MTT: 3-(4,5-dimethylthiazol-2-yl)-2,5-diphenyltetrazolium bromide
PBS: phosphate-buffered saline
TUNEL: terminal deoxynucleotidyl transferase-mediated dUTP nick end labeling
